# Molecular Research on Plasmodium Infection and Immunity

**DOI:** 10.3390/ijms25074133

**Published:** 2024-04-08

**Authors:** Jean-Paul Coutelier, Sylviane Pied

**Affiliations:** 1 The Unit of Experimental Medicine, de Duve Institute of Cellular Pathology, Université Catholique de Louvain, 1200 Bruxelles, Belgium; 2 CNRS UMR 9017-INSERM U1019, Center for Infection and Immunity of Lille—CIIL, Institut Pasteur de Lille, Université de Lille, F-59019 Lille, France; sylviane.pied@pasteur-lille.fr

The WHO’s global strategy for malaria targets a reduction of at least 90% of both incidence and mortality rates for 2030 [1]. However, in 2022, 249 million malaria cases and 608,000 deaths were reported, mostly on the African continent [2]. Vector control through insecticide-treated nets and indoor residual spraying are clearly not sufficient to eradicate the disease. Challenges that must be addressed include *Anopheles* mosquitoes’ resistance to insecticides, the effect of global warming on vector distribution, local reluctance to apply vector control strategies, and *Plasmodium* antimalarial drug resistance. Therefore, it is necessary to better understand *Anopheles’* biology, to improve diagnostic methods, and to target the pathogenic mechanisms leading to severe malaria cases in order to at least decrease the lethal burden of the infection.

Mutations of key parasite genes have allowed the emergence of *Plasmodium* resistance to antimalarial drugs [3,4]. Moreover, migrations of asymptomatic carriers are responsible for the reintroduction of parasites, including drug-resistant mutants, in countries that had previously eliminated malaria [5]. Understanding the relationships between host migration and *Plasmodium* dissemination is important in order to better define global approaches to eliminate the parasite, an issue that is addressed in terms of birds in this Special Issue by Huang et al. [6].

A key strategy for successfully decreasing *Plasmodium* transmission and providing the best treatment is to detect infected individuals as soon as possible [7,8]. To achieve this, Frickmann et al. discuss metagenomic sequencing as an alternative diagnosis approach, especially in non-endemic settings [9], and Calderaro et al. review the methods of malaria diagnosis in non-endemic areas [10].

The clinical presentations of malaria range from asymptomatic to severe illness leading to death from anaemia or neurologic disease. The balance between pro- and anti-inflammatory cytokine responses plays a critical role in the outcome of *Plasmodium* infection [11]. Here, the production of TGF-b and of IL-9 in patients with distinct malaria clinical presentations is analysed by Ndoricyimpaye et al. [12], and the involvement of the IL-33/ST2 pathway in cerebral malaria is reviewed by Glineur et al. [13]. Moreover, neutrophils might also play a role in the development of cerebral malaria, as analysed in a mouse model by Freire-Antunes at al. [14].

Successful clearance of *Plasmodium* and recovery from clinical malaria are dependent on the orchestration of a complex variety of cellular and molecular immune responses. Although key effector mediators include natural killer cells, helper and cytolytic T lymphocytes, and antibody-producing B lymphocytes, the importance of other cells, especially from the innate immune system has recently been shown [15]. IFN-γ is one of the major molecules responsible for appropriate immune responses. Here, Buendia-Gonzalez et al. report on a dehydroepiandrosterone-induced sexual dimorphism in these immune responses that may account for differences in the clinical outcome of men and women infected with *Plasmodium* [16].

Vaccine development should also strongly contribute to the fight against malaria. The most advanced vaccine so far is the RTS,S, targeting the *P. falciparum* circumsporozoide protein, which has already been distributed on a large scale in three African countries [17,18]. However, several other vaccines are currently under development [19]. In this issue, da Silva Matos et al. analyse the potential of RMC-1, a multistage chimeric protein, to become a candidate vaccine against *P. vivax* [20].

Through modulation of the host immune microenvironment, *Plasmodium* may also modulate the course of concomitant diseases that were not initially triggered by the parasite. For instance, *Plasmodium* infection has been associated with a lower prevalence of atopy and allergy-related disease [21], has been shown to affect cancer growth in diverse ways [22,23], and may also facilitate HIV-1 replication through activation of CD4+ T cells [24]. In this Issue, Soe et al. report on the exacerbating effect of mouse *Plasmodium* infection on a concomitant endotoxin shock [25].

Together therefore, the articles in this Special Issue introduce a new understanding of the epidemiology of *Plasmodium* infection, the diagnosis methods, the pathogenic consequences of diverse anti-*Plasmodium* immune responses, and the indirect pathogenic mechanisms triggered by the infection.
